# More AI, Less Care-Seeking? A National Survey Experiment on the Impact of AI Intensity on Patient Care-Seeking Intention in Chinese Family Doctor Services

**DOI:** 10.3390/healthcare14081022

**Published:** 2026-04-13

**Authors:** Feng Jiang, Shengtian Hou, Qianqian Huang, Ruiping Zhao, Yi-Lang Tang

**Affiliations:** 1 School of International and Public Affairs, Shanghai Jiao Tong University, Shanghai 200230, China; fengjiang@sjtu.edu.cn; 2 Institute of Healthy Yangtze River Delta, Shanghai Jiao Tong University, Shanghai 200230, China; 3 Faculty of Health and Wellness, City University of Macau, Macao, China; sthou@cityu.edu.mo; 4 Peking University First Hospital, Beijing 100034, China; 5 Department of Psychiatry and Behavioral Sciences, Emory University School of Medicine, Atlanta, GA 30322, USA; ytang5@emory.edu; 6 Mental Health Service Line, Atlanta VA Medical Center, Decatur, GA 30033, USA

**Keywords:** primary care, artificial intelligence, large language models, care-seeking intention, survey experiment, China

## Abstract

**Background:** Artificial intelligence (AI) is increasingly embedded in routine primary care, yet how the levels of integration might affect its acceptability is unknown, especially in relationship-based service models where patients expect visible human stewardship. Prior experimental studies often treat AI adoption as a binary condition, leaving the “intensity gradient” of automation and the role of model specialization under-explored. We examine whether increasing AI integration in the clinical encounter erodes patients’ intention to seek care from family doctors in China, and whether labeling the AI as a medical-specific model buffers such erosion. **Methods:** We conducted a nationwide online survey experiment in China (N = 2790). Participants were randomly assigned to vignettes that varied by (i) the level of AI integration (low, medium, high) and (ii) the AI type (general-purpose vs. medical-specific large language model), with a human-only care scenario as a reference. Care-seeking intention from family doctors was assessed immediately after exposure. We estimated treatment effects using OLS regression with heteroskedasticity-robust standard errors, and examined the buffering hypothesis through an interaction term between AI integration intensity and AI type. **Results:** Care-seeking intention declined steadily as AI integration increased (*p* < 0.001), with the sharpest drop under high-intensity AI integration where clinical decisions were delegated to the AI system. Across all intensity levels, framing the system as a medical-specific AI consistently resulted in higher care-seeking intention than a general-purpose model. However, the interaction between AI intensity and the AI type was not statistically significant (*p* = 0.508). **Conclusions:** Patient acceptance of AI in primary care depends not only on whether AI is involved, but on how deeply AI is positioned in the encounter. Medical-specific AI labeling may enhance acceptance across all AI integration levels. The findings underscore the need to preserve human clinical agency in AI-embedded primary care. The results contribute to research on healthcare systems, digital health, and AI–patient interaction.

## 1. Introduction

Primary-care systems earn their legitimacy less through spectacular interventions than through routine, low-visibility work—triage, continuity, and the long arc of accompaniment that keeps people from falling through institutional cracks [1]. Across comparative settings, stronger primary care has repeatedly been linked to better population health and more equitable outcomes, even when the fiscal story is complicated [2]. What quietly binds these functions together is not simply access but confidence. When patients trust the professional standing across the desk, they disclose more, adhere more, and report better experiences, effects that accumulate into measurable health differences [3]. China’s contract-based Family Doctor Services (FDS) were built precisely to reposition the system away from a hospital-centric, treatment-first logic and toward earlier intervention and sustained health management, yet persistent gaps in primary-care quality and coordination mean that this institutional wager remains unfinished business [4,5].

Into this relational infrastructure, artificial intelligence, especially large language models (LLMs), arrives with an alluring managerial promise: faster sensemaking, smoother workflows, and help with the follow-up labor that primary care chronically struggles with [6]. But patients rarely treat AI as neutral “plumbing”, as surveys and mixed-method syntheses show enthusiasm braided with reluctance, and a recurring preference that humans remain visibly in charge [7,8]. The behavioral mechanism is familiar that once an algorithm is seen to err, people recoil disproportionately, and when automation is interpreted as substituting for a clinician’s attention to the patient’s particularities, resistance hardens even if performance is high [9,10].

Despite growing interest in AI-assisted healthcare, a critical gap remains in understanding how the degree of AI integration affects patient acceptance. Prior experimental studies have predominantly treated AI adoption as a binary condition (AI vs. no AI), overlooking the nuanced effects of gradual AI integration across different intensity levels [7,10]. Recent work on LLMs in clinical settings has examined diagnostic accuracy and workflow efficiency [11,12,13,14,15], yet patient behavioral responses to varying degrees of AI delegation remain underexplored. Furthermore, whether domain-specific AI tools (e.g., medical-grade LLMs) might mitigate patient resistance compared to general-purpose models has not been systematically tested. This study addresses these gaps by experimentally manipulating both AI intensity and AI specialization framing within China’s primary care context.

This study integrates three theoretical perspectives to understand how AI intensity and AI type jointly shape patients’ care-seeking intention. First, algorithm aversion theory suggests that people exhibit resistance to algorithmic decision-making, particularly in high-stakes domains like healthcare [9,10]. This aversion intensifies as AI becomes more central to decision-making, implying a dose–response relationship between AI intensity and patient resistance. Second, trust theory posits that in relationship-based primary care systems, trust is built on perceived physician competence, benevolence, and agency [3,16]. AI integration challenges perceptions of physician agency, while AI type may influence perceived competence. Third, the Technology Acceptance Model (TAM) predicts that perceived usefulness and ease of use drive technology adoption [17]. However, TAM may not fully capture the relational and trust-based concerns central to primary care, where patients value continuity and personal attention alongside efficiency [16].

From this theoretical foundation, we derive two primary research questions: (1) When AI intensity is experimentally increased from low to high, does care-seeking intention decline? (2) Does framing the AI tool as a medical-specific (versus general-purpose) LLM attenuate resistance by signaling domain competence? We test the following hypotheses:

**H1:**  *As AI intensity increases, patients’ care-seeking intention decreases.*

**H2:**  *Medical LLMs will elicit higher care-seeking intention compared to General LLMs.*

**H3:**  *The negative effect of AI intensity on care-seeking intention will be attenuated by Medical LLMs compared to General LLMs.*

By jointly manipulating integration intensity and model specialization framing, we move the literature beyond binary “AI vs. no-AI” adoption and contribute a testable delegation theory: (a) patient acceptance follows a nonlinear intensity–response function with an empirically identifiable tipping point where perceived clinical discretion shifts away from the physician; and (b) medical-specific credential cues partially offset that tipping by stabilizing competence-based trust, which thereby re-specifying algorithm aversion in healthcare as a problem of authority allocation and accountability visibility, not merely accuracy evaluation.

## 2. Materials and Methods

### 2.1. Study Design

We used a factorial survey experiment with a between-subjects assignment strategy [18,19], primarily because it lets respondents make choices in decision vignettes that resemble real clinical encounters while keeping the informational environment tightly standardized. That combination, ecological plausibility on the surface, experimental control underneath, reduces confounding and supports causal claims about how AI features shape patient responses. In design terms, scenarios varied along two dimensions (AI type: general-purpose vs. medical; integration intensity: low, medium, high), and we added a human-only benchmark condition, yielding seven unique arms in total. Each participant was randomly allocated to one arm, so the factors remained orthogonal by construction.

### 2.2. Experimental Stimuli Construction

All respondents were presented with the same core vignette; they imagined themselves seeking help from a family doctor for a persistent headache. To keep competence-based trust from drifting across conditions, the clinician was described in identical terms each time: “Dr. Zhang, a family doctor with 15 years of clinical experience.” The experimental manipulations focused exclusively on the role and configuration of AI in the clinical encounter.

AI intensity (three levels). We operationalized intensity by varying how central the AI was to the consultation and how much decision authority appeared to shift away from the physician.

Low intensity: AI is portrayed as a back-end support tool used for administrative or organizational tasks (for example, sorting and organizing the patient’s health records). The doctor clearly signals that clinical judgment remains fully human-led (“I will make the decision”).

Medium intensity: AI is positioned as an analytic aid that generates diagnostic reasoning and recommendations. Here, the doctor takes on a checking role, recognizing the AI’s argument while insisting on independent verification (“The AI’s suggestion makes sense, but I need to confirm it”).

High intensity: AI is framed as the dominant decision agent. The doctor explicitly defers to the system’s conclusion and implements an AI-generated plan with minimal reinterpretation (“I decided to follow the AI’s plan completely”).

Two AI Types: (1) General-Purpose Large Language Model (LLM): Labeled as “General Large Models” (e.g., DeepSeek V3, ChatGPT 4). This represents a generic epistemic agent without specific medical institutional endorsement. (2) Medical-Specific LLM: Labeled as “Medical Large Models” (e.g., Shenzhou Medical AI, Xinghuo Medical AI, examples for respondents’ comprehension) [20,21]. This represents an agent that theoretically inherits authority through professional labeling and linkage to medical knowledge bases.

Control Group (The Human Baseline): A pure human-agency scenario where Dr. Zhang explicitly states, “I will rely solely on personal experience and manual review”.

A summary of all experimental scenarios is provided in Table 1.

### 2.3. Participants

Participants were eligible if they met four conditions: they were 18 years of age or older; they held Chinese nationality and lived in China permanently; they were able to complete an online questionnaire; and they could understand the meaning of each item in the survey. Individuals were excluded if they could not communicate effectively or if practical difficulties prevented them from completing the questionnaire.

### 2.4. Data Collection

Data collection ran from 20 June to 10 July 2025, using a snowball recruitment strategy. Survey notices were circulated through community bulletin boards and online forums, with the link embedded so that initial respondents could both participate and pass it on within their networks. Responses were administered and returned via the online survey platform *Wenjuanxing* (www.wjx.cn), and recruitment was intentionally broad, drawing participants from 31 provinces (autonomous regions and municipalities) across mainland China.

To reduce repeat participation, the platform restricted submissions to one response per IP address. Participation was voluntary and anonymous, and every respondent reviewed the consent information and indicated informed consent before proceeding.

Minimum sample requirements were derived from Cochran’s standard formula (SS = Z^2^ · P · (1 − P)/d^2^) [22]. With a 95% confidence level (Z = 1.96), a deliberately tight error tolerance of 2% (d = 0.02), and the conventional “maximum variance” assumption (*p* = 0.50), the implied baseline sample was 2401.

Online surveys rarely yield a fully usable dataset. Attention checks, straight-lining, and other quality screens typically remove a non-trivial share of submissions, so we budgeted for roughly 10% invalid responses and inflated the target accordingly to 2668 (2401/0.9). Beyond supporting national coverage, this sample size is also suited to the factorial experiment’s needs, providing adequate power to detect relatively small differences across scenario conditions.

### 2.5. Measures

Patient care-seeking intention was assessed with a single item: “I am willing to continue being treated by this doctor” [10]. Participants rated their agreement on a seven-point Likert scale, ranging from 1 (“strongly disagree”) to 7 (“strongly agree”) [23]. Single-item measures of behavioral intention have been shown to be appropriate and reliable in experimental vignette studies.

To capture baseline differences in technology receptivity, we included three covariates. AI usage frequency was recorded on a five-point scale from “never” to “daily”. AI knowledge was measured via self-rated familiarity with medical AI, also on a five-point scale spanning “completely unaware” to “mastery of technical principles”. Finally, technological attitude tapped respondents’ general orientation toward AI development, ranging from “strong concern about risks” to “firm belief that technology solves everything” (five-point scale).

Basic sociodemographic information was collected based on the literature review [24].

### 2.6. Procedure

The data collection followed a strict three-phase protocol to establish baseline characteristics before introducing the experimental stimulus.

Phase 1: Baseline assessment. After entering the online platform and indicating informed consent, respondents completed a background module before seeing any experimental materials. This section captured standard sociodemographic characteristics (gender, age, education, and income) along with AI-related covariates—how often they use AI tools, how familiar they consider themselves with medical AI, and their broader orientation toward technological development. Measuring these factors upfront helped keep “trait-like” technology dispositions from being shaped by, or conflated with, the vignette exposure that followed.

Phase 2: Randomized intervention. Once the baseline section was completed, the survey system randomly allocated each respondent to one of the seven vignette conditions. Participants were asked to read the scenario as if they were experiencing the consultation themselves, with the encounter differing only in the level and form of AI integration (or, in the control arm, no AI at all).

Phase 3: Outcome measurement. Right after the vignette, respondents reported their care-seeking intention, anchored to the particular doctor–AI arrangement described in the scenario they had just read.

### 2.7. Data Analysis

Data processing and statistical analyses were carried out in Stata 18.0 (StataCorp LLC, College Station, TX, USA). We began by profiling the sample with descriptive statistics. For continuous measures, results are reported as means and standard deviations (SD); for categorical variables, we present counts and percentages.

Second, we checked whether random assignments actually produced comparable groups across the seven conditions. For continuous variables (such as age and AI use frequency), we compared means using one-way ANOVA; for categorical variables (such as gender), we relied on chi-square tests. These balance tests were used to flag any systematic differences between arms that might indicate a failure of randomization.

Third, we evaluated the hypotheses about how AI intensity and AI type shape care-seeking intention using OLS linear regression. The modelling strategy unfolded in stages. We estimated five OLS regression models with heteroskedasticity-robust (HC3) standard errors to examine the effects of AI intensity, AI type, and their interaction on care-seeking intention.

Model 1 examined the main effect of AI intensity using the full sample, with dummy variables for low, medium, and high intensity (reference: human-only).

Model 2 examined the main effect of AI type within the AI scenarios with a binary indicator for medical LLM (reference: general LLM).

Model 3 provided a condition-by-condition specification treating each of the six AI vignettes as a distinct exposure (reference: human-only), allowing the combined effects of intensity and type to emerge.

Model 4 was the primary model for testing the interaction between AI intensity and AI type. Using the AI sample only, this model included AI intensity (continuous: 1 = low, 2 = medium, 3 = high), LLM type (binary: 0 = general, 1 = medical), and their interaction term (AI intensity × LLM type).

Model 5 extended the interaction specification to the full sample. Model comparison used Akaike Information Criterion (AIC), with lower values indicating better fit. All models controlled for the full set of demographic and AI-related covariates listed above. Statistical significance was assessed at α = 0.05.

To make the joint pattern of AI intensity and model type easier to interpret, we graphed predicted values of care-seeking intention for each condition using predictive margins and added 95% confidence intervals to show uncertainty around the estimates. All hypothesis tests were two-sided, with statistical significance evaluated at *p* < 0.05.

## 3. Results

A total of 3020 participants were initially recruited. After data screening, 230 responses (7.6%) were excluded based on the following criteria: (1) Implausible demographic values (e.g., age ≥ 120 years, n = 12); (2) Logical contradictions in survey responses (e.g., selecting mutually exclusive options, n = 47); (3) Failed attention checks embedded within the vignettes (completion time < 3 min, n = 171). The final analytical sample comprised 2790 participants. Comparison of excluded versus retained participants revealed no significant differences in demographic composition (gender: χ^2^ = 1.23, *p* = 0.27; age group: χ^2^ = 3.45, *p* = 0.33), suggesting that exclusion criteria did not disproportionately affect any particular subgroup.

Table 1 presents the sociodemographic profile and key variable characteristics of the study sample. The participants were middle-aged, with over two-thirds being female (67.24%), and 79.25% residing in urban areas. The educational attainment of the samples was relatively high, with 69.35% holding a bachelor’s degree. Regarding employment and economic status, 60.07% were currently employed, and the largest proportion of participants (51.97%) were covered by the Urban Employee Basic Medical Insurance.

In terms of technology familiarity, participants reported a moderate frequency of AI usage (3.07 ± 1.16) and a moderate level of self-rated familiarity with medical AI (2.76 ± 0.76). The overall attitudes toward AI development were slightly positive (3.01 ± 0.99). Table 2 shows the details.

The baseline level of care-seeking intention in the overall sample was relatively high (5.27 ± 1.59), indicating a general willingness to engage with family doctor services.

As shown in Table 3, no significant differences were found in the baseline characteristics, confirming that the randomization was successful and ensuring that subsequent treatment effects were unlikely to be confounded by pre-existing group differences.

After adjusting for the full set of covariates, Table 4 reports the OLS estimates for how AI integration relates to care-seeking intention.

To examine the effects of AI intensity and AI type on care-seeking intention, we estimated five models with robust standard errors. Model 1 examined AI intensity effects using the full sample (N = 2790). Relative to the human-only baseline, low (β = −0.682, *p* < 0.001), medium (β = −1.053, *p* < 0.001), and high (β = −1.267, *p* < 0.001) AI intensity were each associated with significantly lower care-seeking intention, demonstrating a graded negative effect.

Model 2 focused on AI-type effects within the AI conditions only (N = 2393), revealing that medical LLMs elicited marginally higher care-seeking intention than general LLMs (β = 0.152, *p* = 0.086), though this difference did not reach conventional statistical significance.

Model 3 provided a condition-by-condition specification, treating each of the six AI vignettes as a distinct exposure. Results showed that care-seeking intention was highest in the human-only condition and declined monotonically with increasing AI intensity, with the steepest decline observed for general LLMs at high intensity (β = −1.341, *p* < 0.001).

Critically, Model 4 formally tested the interaction between AI intensity and LLM type. Using the AI-only sample (N = 2393), this model included AI intensity (coded as 1 = low, 2 = medium, 3 = high), LLM type (0 = general, 1 = medical), and their interaction term. The main effect of AI intensity was significantly negative (β = −0.521, *p* < 0.001), indicating that each unit increase in intensity reduced care-seeking intention by 0.521 points for general LLMs. The interaction term showed a positive coefficient (β = 0.052, *p* = 0.508), suggesting that medical LLMs may attenuate the negative effect of AI intensity, although this interaction was not statistically significant at the conventional 0.05 level.

The magnitude of the coefficient implies a 10.0% buffering effect. For medical LLMs, the intensity effect was −0.469 (i.e., −0.521 + 0.052), compared to −0.521 for general LLMs.

Model comparison showed that the interaction model (Model 4) achieved the lowest AIC (8754.2) among specifications tested on the AI-only sample, suggesting improved model fit with the inclusion of the interaction term.

Model 5 extended the interaction analysis to the full sample, yielding consistent patterns.

Across all models, several control variables emerged as significant predictors. Positive attitudes toward AI technology development (β = 1.783–1.802, *p* < 0.001 across models) and higher AI tool usage frequency (β = 0.080–0.106, *p* < 0.05) were consistently associated with higher care-seeking intention.

**Figure 1:**
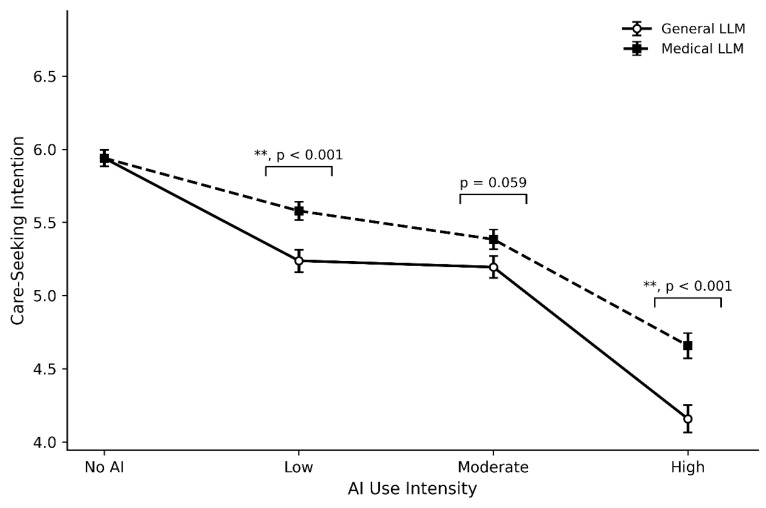
AI intensity and care-seeking intention. Note: ** means that the *p*-value is less than 0.001.

As shown in Figure 1, the human-only control elicited the strongest care-seeking intention (M = 5.94, SD = 1.15). Once AI enters the consultation, care-seeking intention declines in a graded manner, with the pattern diverging across AI types. For the general purpose LLM, intention drops under low-intensity integration (M = 5.24, SD = 1.53), shows minimal change at the medium level (M = 5.19, SD = 1.49), and then falls sharply when integration becomes high (M = 4.16, SD = 1.90).

The medical LLM follows a notably softer trajectory. Intention decreases from low (M = 5.58, SD = 1.27) to medium (M = 5.38, SD = 1.34) to high intensity (M = 4.66, SD = 1.71), but at every matched intensity level, medical LLMs elicit higher care-seeking intention than general LLMs.

Conditional effects analysis using independent-samples *t*-tests with pooled standard errors revealed that Medical LLMs provided buffering effects across all three intensity levels, though statistical significance varied. At low intensity, Medical LLMs buffered 48.6% of the negative effect (Δ = 0.34 points, SE_pooled = 0.10, *t* = 3.40, *p* < 0.001), representing a substantial and highly significant recovery, nearly half of the intention loss from AI integration was offset.

At medium intensity, the buffering effect diminished to 25.3% (Δ = 0.19 points, SE_pooled = 0.10, *t* = 1.92, *p* = 0.059), failing to reach conventional statistical significance at the 0.05 level, suggesting weaker differentiation between LLM types at this AI intensity level.

Notably, at high intensity, the buffering effect recovered to 28.1% (Δ = 0.50 points, SE_pooled = 0.11, *t* = 4.42, *p* < 0.001), demonstrating the largest *t*-statistic and a highly significant advantage for Medical LLMs even when AI assumes primary decision-making authority.

This non-linear pattern suggests that Medical LLMs’ buffering benefit operates through different mechanisms across the intensity gradient, with the most reliable effects at the extremes of the intensity spectrum.

## 4. Discussion

Using a nationwide survey experiment that parameterizes integration intensity rather than treating adoption as a binary state, we show that care-seeking intention follows a nonlinear delegation curve, comparatively stable at low levels of AI integration, then dropping sharply once AI becomes the primary agent of decision-making.

For healthcare-systems research, this identifies a concrete mechanism through which digital modernization can exact a relational price: when clinician agency becomes less legible, trust weakens in ways that translate into altered utilization intention [3]. For digital-health scholarship, the results refine the dominant productivity narrative by specifying a design-relevant boundary condition: scaling AI is not monotonic in social acceptability, and the point of failure is tied to perceived substitution rather than computational presence [6,25]. Finally, for AI–patient interaction, our manipulation of model framing indicates that medical-specific credential cues partially buffer the tipping point, re-specifying medical AI resistance as a problem of authority allocation and accountability visibility as much as one of accuracy evaluation—an extension of algorithm aversion and resistance arguments into routine, relationship-based care [9,10].

The pattern we observed, lower care-seeking intention as AI becomes more deeply embedded in the encounter, maps closely onto what the “algorithm aversion” literature has been arguing for years [9]. Longoni et al. (2019), together with Chen and Cui (2025), describe a stubborn form of patient resistance to automated medical agents that is rooted in uniqueness neglect, the suspicion that algorithmic outputs are calibrated to population-level regularities and therefore gloss over the patient’s own biological particularities and lived, biographical contingencies [10,24]. In other words, once clinical judgment is seen as being relocated from a person to a system, the promise of individualized care can feel thinner, even when the technical performance is presumed to be strong. Our results do more than echo prior findings; they underline a basic coupling in medical interaction between personalization and human agency, where being “seen as an individual” is not merely an informational achievement but a relational one.

At the same time, our evidence sits uneasily with a more TAM-oriented stream of work, where adoption is largely framed as a function of perceived usefulness and performance gains, diagnostic speed, accuracy, and convenience being treated as the decisive levers of acceptance [26,27]. In many of those studies, patient receptivity looks relatively high, and the implicit claim is straightforward: if AI delivers better outcomes, patients will come along for the ride.

Why do we see something less sanguine? The difference is not simply methodological; it is ecological. A substantial share of the “optimistic” evidence comes from settings where the task is tightly bounded and comparatively objective, such as radiology reads, dermatology screening, or other image-interpretation problems, or from tertiary-care environments where patients are primarily seeking a correct answer as efficiently as possible [28]. That is a service world in which the interaction is closer to a transaction about accuracy.

FDS operate under a different institutional logic. Here, the expected value of care is carried by continuity, stewardship over time, and an affective sense of being accompanied, not merely by throughput in clinical decision-making. In such a relational setting, efficiency can register as a secondary virtue, and the introduction of AI (especially when it becomes highly visible or directive) may be read as a thinning of interpersonal warmth. The marginal utility of speed is often dominated by the perceived loss of human presence, which helps explain why intention drops more sharply in our context than TAM-based accounts would predict.

In addition, much of the experimental evidence to date has treated AI use as a yes/no switch, so the outcome is almost predetermined, and respondents tend to pick the human option [29]. We tried to move past that blunt contrast by modelling AI integration as a spectrum, ranging from low to medium to high integration. That shift matters because it allows a more informative inference than “people prefer humans”.

What emerges from the intensity gradient is a tolerance threshold. Patients are not necessarily objecting to AI as a tool; what draws resistance is the perception that the tool is starting to replace the clinician’s role rather than supporting it. Light and medium AI intensity levels appear broadly tolerable, whereas heavy integration, where decision authority visibly migrates toward the system, corresponds to a sharper, trust-related break. Framed this way, the practical implication is clearer: the Human-in-the-Loop principle is not just a slogan, but a boundary condition, and our design helps locate where that boundary is likely to sit.

Even though deeper AI integration tends to pull care-seeking intention downward overall, the medical LLM consistently holds up better than a general-purpose model in preserving patients’ willingness to continue care.

This fits well with what source credibility theory would lead us to expect in information systems settings: when people evaluate automated advice, they often lean on quick, heuristic signals of expertise rather than auditing the underlying logic line by line [30,31]. In our scenarios, framing the agent as a medical model, explicitly trained on clinical guidelines and authoritative knowledge, appears to supply that credibility cue. The tool, in effect, borrows institutional legitimacy from the domain it claims to represent, which can soften (though not eliminate) the algorithm aversion that tends to attach to generic, non-specialized systems.

Although conditional effects analysis suggested potential buffering effects, the effect did not reach statistical significance. Several factors may explain this non-significance, including limited statistical power to detect small interaction effects, measurement constraints of the single-item outcome, and potential ceiling effects at low intensity levels. Future research with larger samples should re-examine this interaction. These post hoc analyses are exploratory in nature and should not be interpreted as confirming the buffering hypothesis. The conditional effects were neither pre-registered nor derived from a priori theoretical thresholds; the distinction between low-medium and medium-high intensity was determined post hoc based on our experimental design. Consequently, we cannot conclude that Medical LLMs significantly buffer the negative effects of AI intensity on care-seeking intention. Replication with larger samples and pre-registered analysis plans is necessary to adequately test this hypothesis.

This study makes some theoretical contributions. First, we synthesize algorithm aversion, trust theory, and TAM, and demonstrate that no single theoretical perspective fully captures patient responses to AI-assisted healthcare. Second, the interaction between AI intensity and AI type challenged the assumption that specialized knowledge always enhances trust. Third, our findings contribute to the broader literature on conditional effects in healthcare technology adoption. The diminishing effect of Medical LLMs’ advantage at higher AI intensity suggests that the benefits of specialized AI systems are not universal but are bound by implementation context. This has implications for both theory development, which must account for boundary conditions, and practice, which must consider implementation strategies that optimize the effectiveness of AI technologies.

The practical implications of our findings are best read as sociotechnical design lessons rather than as country-bound prescriptions. Once AI is embedded in primary care, what is being governed is the visibility of discretion—who appears to decide, who can be questioned, and who is answerable when things go wrong [16,32]. In that light, the intensity gradient is not merely an implementation detail; it functions as a risk lever, implying that scaling AI in frontline care may need to be staged and reversible, with explicit attention to the point at which patients interpret support as substitution and begin to withdraw [6,10]. Put differently, human-in-the-loop is not only a technical safeguard but a legitimacy device. Oversight must be legible if systems want efficiency gains without eroding the relational infrastructure that brings patients into care in the first place [16,32]. The buffering effect of medical-specific framing also has a governance reading. Because resistance is often triggered by perceived authority displacement rather than by a simple dislike of machines, risk governance needs to treat patient experience and equity impacts as first-order outcomes, so that algorithmic deployment does not quietly reallocate burdens to those least able to contest or navigate the system [9,25].

There are several limitations in this study. First, although the experimental structure strengthens internal validity, our scenarios were delivered as text vignettes, which inevitably limits ecological realism. In actual clinical settings, physicians rarely inform patients that they are completely relying on artificial intelligence for diagnosis or treatment recommendations. What respondents say they would do in a hypothetical scenario may not fully match what they actually do in a clinic [33]. While scenario-based experimental designs offer advantages for causal inference by isolating specific variables of interest, they inherently sacrifice some external validity. Future research should incorporate multiple methodological approaches, including field experiments in actual clinical settings, high-fidelity clinical simulations with standardized patients, and observational studies in real healthcare contexts, to enhance the generalizability of our findings and better capture how patients respond to varying degrees and forms of AI disclosure in authentic medical encounters.

Second, measurement of AI intensity was based on three discrete levels (low, medium, high), which may not capture the full continuum of AI integration in primary care settings. Future research could use more granular measures of AI intensity, such as continuous scales or more detailed task-level assessments, to better characterize the relationship between AI integration and patient outcomes.

Third, our study focused on care-seeking intention as the primary outcome using a single Likert-scale item, but other outcomes, such as treatment adherence, patient satisfaction, or health outcomes, may also be influenced by the interaction between AI intensity and LLM type. Future research should examine these outcomes to provide a more comprehensive understanding of AI’s impact on healthcare delivery. At the same time, this study did not directly measure patients’ perceptions of AI hallucinations. Hallucination risk represents a critical concern in healthcare AI applications. Future research should examine how explicit discussion of hallucination probabilities affects patient acceptance of AI-assisted care. However, the modest R^2^ values across models indicate that a substantial proportion of variance in care-seeking intention remains unexplained by the observed variables. Future research should incorporate additional predictors to capture the full complexity of patient responses to AI-assisted healthcare.

Fourth, our use of IP-address restriction to prevent duplicate responses could prevent multiple submissions from shared IP addresses, where legitimate but distinct respondents might be incorrectly blocked. Additionally, mobile devices using dynamic IP addresses complicate IP-based restrictions. Future studies should consider complementary quality control measures.

Fifth, the sampling strategy may limit the generalizability of our findings. Although our sample included respondents from all 31 provinces in mainland China, the demographic profile shows higher proportions of urban residents and individuals with bachelor’s degrees or higher education compared to national averages. This distribution likely reflects the digital nature of recruitment, which inherently biases the sample toward respondents with greater digital literacy and more exposure to AI technologies through education and urban living environments.

Finally, the study is situated within a particular institutional and cultural setting, where continuity and interpersonal trust are not peripheral features but defining elements of the model. Cross-national and cross-cultural comparisons would be valuable for identifying the boundary conditions under which medical AI is more readily accepted, and where it is most likely to provoke resistance.

## 5. Conclusions

Our findings indicate that the pivotal issue for patient acceptance is not whether AI is integrated in primary care, but how the intensity of its integration reshapes accountability and perceived decision-making role. As AI shifts from supportive functions to a dominant decision-maker in clinical scenarios, patients’ care-seeking intention declines markedly, and medical-specific labeling provides only a partial buffer. Taken together, the evidence suggests that rising AI intensity shifts the trust architecture of relationship-based care by altering who is perceived to decide and who can be held accountable. This underscores the need for system designers and policymakers to ensure that human clinical agency remains both substantive and visible as AI capabilities expand.

## Figures and Tables

**Table 1:** Summary of Experimental Scenarios.

Condition	AI Type	Integration Intensity	Physician’s Role Description
Group 1	No	No	Relies solely on experience; explicitly rejects AI usage
Group 2	General LLM	Low	Uses AI to organize records; the physician retains full decision power
Group 3	General LLM	Medium	Uses AI for analysis; the physician validates and verifies suggestions
Group 4	General LLM	High	Relies on AI for diagnosis; the physician executes AI’s plan
Group 5	Medical LLM	Low	Uses AI to highlight abnormal indicators; the physician leads the diagnosis
Group 6	Medical LLM	Medium	Uses AI for guideline-based suggestions; the physician evaluates the validity
Group 7	Medical LLM	High	Relies on AI linked to authoritative databases; the physician complies with the AI report

**Table 2:** Sociodemographic characteristics of participants (N = 2790).

Variables	Summary Statistics n(%)
Gender	
Male	914 (32.76)
Female	1876 (67.24)
Location	
Urban	2211 (79.25)
Rural	579 (20.75)
Marriage	
Single	1490 (53.40)
Married (including cohabiting)	1300 (46.59)
Education background	
Less than primary school	23 (0.82)
Junior high school	37 (1.33)
Senior high school/Technical secondary school	111 (3.98)
Junior college	293 (10.50)
Bachelor’s degree	1935 (69.35)
Master’s degree or above	391 (14.01)
Employment status	
Unemployed	1114 (39.93)
Employed	1676 (60.07)
Monthly income (RMB/month)	
≤1000	124 (4.44)
1001–2000	179 (6.42)
2001–3000	292 (10.47)
3001–4000	351 (12.58)
4001–5000	372 (13.33)
5001–6000	331 (11.86)
6001–9000	409 (14.66)
9001–12,000	332 (11.90)
12,001–15,000	192 (6.88)
≥15,001	208 (7.46)
Health insurance coverage	
Self-pay	532 (19.10)
Urban–Rural Resident Basic Medical Insurance	971 (34.80)
Urban Employee Basic Medical Insurance	1287 (46.10)
	mean (SD)
Age	30.50 (9.093)
AI tool usage frequency	3.07 (1.164)
Self-rated familiarity with medical AI	2.76 (0.762)
Attitude toward AI technology development	3.01 (0.988)
Care-seeking intention	5.27 (1.585)

**Table 3:** Balance Check of the 7 groups.

	Group 1 (N = 397)	Group 2 (N = 396)	Group 3 (N = 397)	Group 4 (N = 405)	Group 5 (N = 408)	Group 6 (N = 396)	Group 7 (N = 391)	χ^2^	*p*
Gender (male, %)	35.01	32.32	33.50	32.84	33.82	33.33	28.39	4.709	0.582
Education (primary school, %)	1.26	0.76	1.01	0.99	0.74	0.51	0.51	2.237	0.897
Marriage (Married, %)	47.36	45.71	48.36	43.70	46.57	45.20	49.36	3.587	0.732
Employment (Employed, %)	62.72	60.61	59.45	55.80	59.56	59.09	61.64	4.870	0.561
Income (≤1000, %)	4.79	6.31	4.79	4.94	2.45	2.78	5.12	10.529	0.104
Location (Urban, %)	80.35	77.53	81.61	78.77	78.92	78.54	79.03	2.576	0.860
Insurance (Urban Employee Basic Medical Insurance, %)	48.87	44.95	45.84	39.75	49.75	45.45	48.34	13.74	0.318
	mean							F	*p*
age	30.48	30.15	31.36	29.83	30.82	30.29	30.60	0.815	0.558
AI tool usage frequency	3.22	3.15	3.21	3.10	3.21	3.14	3.21	0.696	0.653
Self-rated familiarity with medical AI	2.68	2.71	2.76	2.65	2.71	2.66	2.71	0.866	0.519
Attitude toward AI technology development	2.90	2.92	2.94	2.82	2.89	2.88	2.92	0.703	0.647

**Table 4:** Factors Associated with Care-seeking Intention.

	Model 1 (Ref: Human-Only, N = 2790)	Model 2 (Ref: General LLM, N = 2393)	Model 3 (Ref: Human-Only, N = 2790)	Model 4 (Ref: Low, General LLM, N = 2393)	Model 5 (Ref: Human-Only, N = 2790)
VARIABLES	Intention	Intention	Intention	Intention	Intention
AI intensity: Low	−0.539 ***				
	(0.076)				
AI intensity: Medium	−0.670 ***				
	(0.079)				
AI intensity: High	−1.536 ***				
	(0.087)				
LLM Type: Medical (vs General)	0.349 ***				
	(0.064)				
AI Intensity				−0.521 ***	−0.535 ***
				(0.058)	(0.034)
LLM Type				0.236	0.202
				(0.155)	(0.115)
AI Intensity × LLM Type Interaction				0.052	0.066
				(0.078)	(0.063)
General LLM-Low			−0.712 ***		
			(0.093)		
General LLM-Medium			−0.787 ***		
			(0.093)		
General LLM-High			−1.754 ***		
			(0.109)		
Medical LLM-Low			−0.371 ***		
			(0.085)		
Medical LLM-Medium			−0.553 ***		
			(0.089)		
Medical LLM-High			−1.313 ***		
			(0.104)		
Gender	0.013	−0.039	0.008	−0.022	0.004
	(0.061)	(0.070)	(0.061)	(0.068)	(0.061)
Age	−0.000	0.041	−0.000	0.027	0.009
	(0.043)	(0.050)	(0.043)	(0.048)	(0.043)
Education background	−0.209 ***	−0.214 ***	−0.208 ***	−0.230 ***	−0.207 ***
	(0.039)	(0.044)	(0.039)	(0.043)	(0.039)
Marriage	0.192 *	0.187	0.190 *	0.210 *	0.186 *
	(0.088)	(0.105)	(0.088)	(0.100)	(0.088)
Employment	−0.057	−0.074	−0.059	−0.075	−0.075
	(0.079)	(0.093)	(0.079)	(0.089)	(0.079)
Monthly income	−0.000	−0.000	−0.000	−0.000	−0.000
	(0.000)	(0.000)	(0.000)	(0.000)	(0.000)
Location	−0.037	−0.050	−0.035	−0.035	−0.027
	(0.073)	(0.083)	(0.073)	(0.081)	(0.073)
Health insurance coverage	0.118 *	0.102	0.106 *	0.090	0.110 *
	(0.047)	(0.054)	(0.046)	(0.052)	(0.047)
AI tool usage frequency	−0.007	−0.001	−0.012	−0.003	−0.011
	(0.029)	(0.034)	(0.029)	(0.033)	(0.029)
Self-rated familiarity with medical AI	0.265 ***	0.308 ***	0.267 ***	0.303 ***	0.271 ***
	(0.041)	(0.048)	(0.041)	(0.047)	(0.041)
Attitude toward AI technology development	0.234 ***	0.265 ***	0.234 ***	0.260 ***	0.235 ***
	(0.033)	(0.036)	(0.033)	(0.036)	(0.033)
Constant	5.098 ***	3.907 ***	5.132 ***	5.035 ***	5.102 ***
	(0.209)	(0.231)	(0.209)	(0.261)	(0.210)
R-squared	0.1666	0.0871	0.1768	0.1492	0.1663
AIC	10,048.4	8918.8	10,020.1	8754.2	10,049.5

Note: *** *p* < 0.001, * *p* < 0.05.

## Data Availability

The data presented in this study are available on request from the corresponding author. Data are not publicly available due to privacy and ethical restrictions.

## Abbreviations

The following abbreviations are used in this manuscript:

AI	Artificial Intelligence
LLMs	Large Language Models
FDS	Family Doctor Services
TAM	Technology Acceptance Model
